# Bond Stress Distribution at the Joint Between Rock Bolts and Grout Under Different Grouting Conditions

**DOI:** 10.3390/ma19173578

**Published:** 2026-08-23

**Authors:** Jianhang Chen, Jie Fang, Yong Zhang, Chenyang Zhu, Pengyu Zhang

**Affiliations:** 1 State Key Laboratory of Water Resource Protection and Utilization in Coal Mining, Beijing 102211, China; 2 School of Energy and Mining Engineering, China University of Mining and Technology (Beijing), Beijing 100083, China; 3 Zhongye Changtian International Engineering Co., Ltd., Changsha 410205, China

**Keywords:** ground controlling, rock anchors, bonding capacity, joint remaining strength

## Abstract

In rock reinforcement systems, bond stress at the joint between rock bolts and grout exerts a crucial influence on determining the bonding capacity of rock bolts. However, much less research has been conducted to study the joint bond stress (JBS) distribution with analytical deduction. Therefore, this paper adopted an analytical model to evaluate JBS distribution. The novelty of this paper is that the JBS distribution under different grouting conditions can be quantitatively studied. Based on variable controlling techniques, the influence of different parameters on JBS distribution state was studied. These studied parameters included joint strength, joint remaining strength and relative slide at joint remaining strength. Results showed that after rock bolts were loaded, the loading process can be basically divided into five different stages. Joint strength exerts a crucial influence on determining the JBS distribution state before the joint fully disconnected. After the joint started disconnecting, larger joint strength led to a slower propagation speed of the maximum JBS. When the remaining strength of the joint increased, after the whole joint deformed plastically, the disconnected length became longer, and frictional resistance in the disconnected section became larger. This led to an increase in the bonding capacity of rock bolts. Compared with joint strength and joint remaining strength, relative slide at joint remaining strength had a slightly smaller influence on JBS distribution. Before the joint disconnected, increasing relative slide at joint remaining strength was likely to improve the propagation speed of the maximum JBS. This study is valuable for further understanding the stress-transferring mechanism in rock reinforcement systems.

## 1. Introduction

Rock reinforcement is an important technique to maintain the stability and safety of underground excavations [1]. Among multiple rock reinforcement techniques, rock bolting is one of the most commonly used methods [2]. After underground excavations are created, rock bolts are used to reinforce the rock mass. Then, rock bolts and the host rock mass become an integral [3]. Materials of rock bolts are usually steel or fibre-reinforced polymers [4]. Therefore, tensile stiffness or tensile strength of rock bolts can be quite high. Then, the tensile capacity of the integrity, which is composed of rock bolts and host rock mass, can be improved apparently. This is more obvious when compared with the tensile capacity of pure host rock mass.

To ensure the integrity of rock bolts and host rock mass, grout plays an important role [5]. In engineering cases, grout material can be cementitious grout or polyester resin [6]. They are injected or pumped into pre-drilled boreholes. Then, after rock bolts are inserted into boreholes, grout can bond rock bolts and host rock mass together. Therefore, in rock reinforcement systems, grout acts as the medium between rock bolts and host rock mass. In fact, grout determines the stress that can be transferred between rock bolts and host rock mass [7].

**Figure 1:**
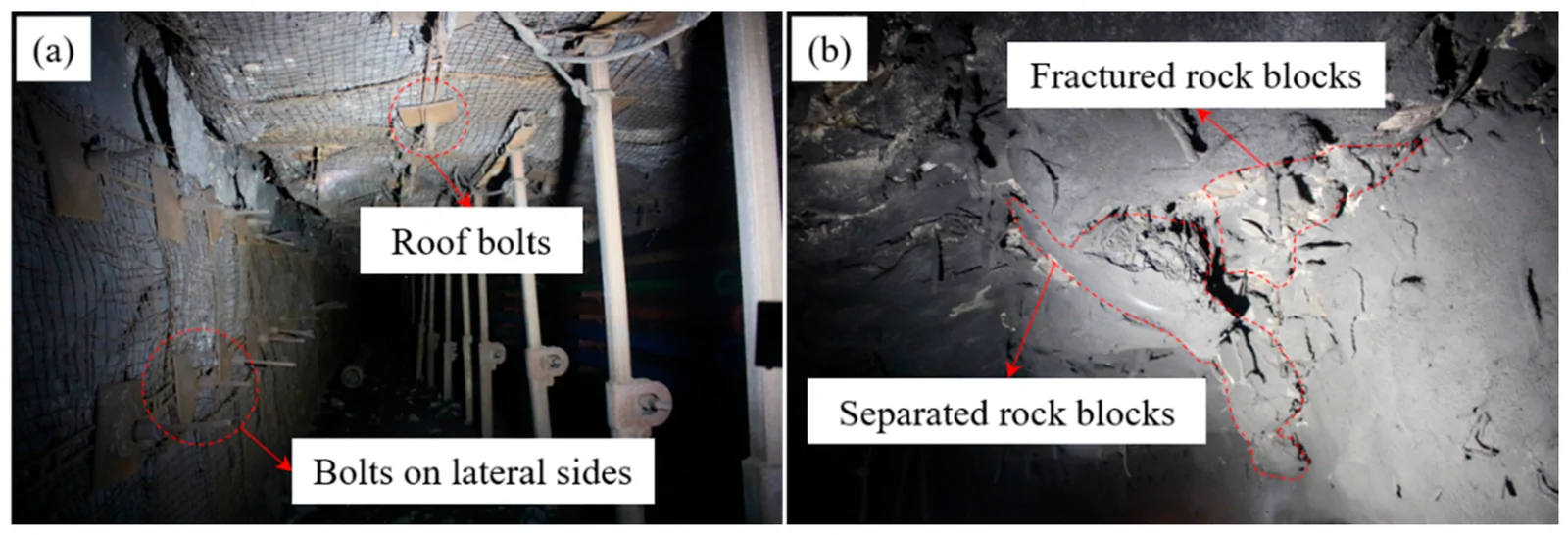
Deformation of underground roadways and collapsing status of rock mass: (**a**) rock bolts were used to reinforce the roof and lateral sides of roadways; (**b**) under the influence of mining activities, rock mass around the roadway roof tended to collapse.

In in situ applications, rock mass is likely to move towards the excavation direction (Figure 1).

Moreover, along the lateral sides of underground excavation, rock mass displacement is larger than that of rock bolts. Therefore, the relative slide between rock bolts and host rock mass induces shear deformation of grout [8]. This leads to the generation of stress in grout.

After stress in grout increases from zero, it cannot increase continuously. This is because the mechanical properties of grout are largely dependent on the mixing proportion of grouting materials, such as water/cement ratio or sand/cement ratio [9]. Therefore, grout has a certain joint strength. Once stress in grout increases to joint strength, grout will undergo plastic deformation. Then, stress in grout starts decreasing. It is noted that once grout fails, stress in grout will not decrease monotonously. This is because after grout fails, intact grout will be split into multiple blocks [10]. During the splitting process, fine and small grout pieces are generated. Moreover, these fine and small grout pieces will fill the gap between split blocks. Because of the filling of these fine and small grout pieces, mechanical interlock between grout blocks will be enhanced. Consequently, after grout failure and grout stress decreases to a certain value, it is likely to become stable. Because of this certain value, it is usually regarded as the joint remaining strength [11].

To simulate this variation process of stress during the grout failure process, previous researchers have used different models, including the two-equation model, three-equation model and non-linear model. Their comparison is tabulated in Table 1.

Among them, the three-equation model is more widely used [12]. The three-equation model is generally composed of three parts. The first part is simulated with a linear increasing equation, which is used to depict the elastic deformation behaviour of grout [13]. Then, the second part is simulated with a linear decreasing equation, which is used to depict the weakening behaviour of grout in the plastic deformation process. The third part is simulated with a constant value, which is used to depict residual shear behaviour of grout in the plastic deformation process.

For in situ underground scenarios, rock bolt length is usually less than three metres [14]. Moreover, with rock bolt length increasing, the relative slide difference between rock bolts and host rock mass can be largely different from the internal end of rock bolts to the external end. Since stress in grout is largely related to the relative slide between rock bolts and host rock mass, there will be apparent stress distribution in grout [15]. Moreover, this stress distribution in grout exerts a crucial influence on determining the bonding capacity of rock bolts.

However, previous research mainly focuses on the macro mechanical response of rock bolting systems. Specifically, more attention is paid to the load–displacement performance of rock bolts. Consequently, there is still a research gap regarding JBS distribution at the internal joint between rock bolts and grout, especially when grouting conditions change. Therefore, in this study, analytical modelling methods are used to study JBS distribution when grouting conditions vary. Compared with previous studies, the main advancement of this paper is that the JBS distribution can be quantitatively studied under varying grouting conditions. This paper is divided into four different parts. First, analytical functions are illustrated. Second, experimental confirmation of the adopted analytical model is explained. Third, the JBS distribution under varying grouting conditions is analysed. Last, the influence of grout properties on JBS distribution is discussed. This study provides a theoretical basis for preventing rock bolting failure disasters in in situ cases.

## 2. Analytical Deduction and Experimental Validation

### 2.1. Analytical Deduction Process

To properly calculate and predict the bonding capacity of rock bolts, the first author of this paper previously developed an analytical method for rock bolting systems [16]. It is commonly accepted that there is a balanced relationship between JBS and tensile stress in rock bolts. In fact, after underground openings are created, without considering rock burst issues, the rock mass around underground openings deforms gradually. This can be regarded as a static loading condition. Then, Equation (1) is obtained.

(1) $$\tau \left(x\right)=\frac{D}{4}\frac{d\sigma_{b}\left(x\right)}{dx}$$

where *τ*: JBS; *x*: x-coordinate from the internal end of rock bolts to the external end; *D*: diameter of rock bolts; and *σ_b_*: tensile stress in rock bolts.

There is a definite relationship between JBS and relative slide. Moreover, in this study, a three-equation model is adopted to calculate JBS [17]. Laboratory and in situ anchorage tests on rock bolts show that after rock bolting systems are loaded, JBS first increases gradually to the peak. Then, JBS decreases gradually. However, when relative slide reaches a limit, JBS tends to be stable. Following this phenomenon, Equations (2)–(4) are used to depict the JBS variation process. However, it should be acknowledged that the current three-equation model has limitations. In reality, after rock bolting systems are loaded, the JBS may not strictly follow a linear relationship with relative slide. Nevertheless, using this three-equation model makes it possible to calculate the JBS with mathematical methods.

(2) $$\tau =\frac{\tau_{p}}{\delta_{p}}\delta \;\left(0\leq \delta \leq \delta_{p}\right)$$

(3) $$\tau =\frac{\tau_{p}\delta_{r}-\tau_{r}\delta_{p}}{\delta_{r}-\delta_{p}}-\frac{\tau_{p}-\tau_{r}}{\delta_{r}-\delta_{p}}\delta \;\left(\delta_{p}<\delta \leq \delta_{r}\right)$$

(4) $$\tau =\tau_{r}\;\left(\delta >\delta_{r}\right)$$

where *τ_p_*: joint strength; *δ_p_*: relative slide at joint strength; *δ*: relative slide; *δ_r_*: relative slide at joint remaining strength; and *τ_r_*: joint remaining strength.

After substituting Equations (2)–(4) into Equation (1), the loading stages of rock bolts can be studied. This study divides the loading process of rock bolts into five loading stages: elastic stage (Stage I), elastic-weakening stage (Stage II), elastic-weakening-disconnecting stage (Stage III), weakening-disconnecting stage (Stage IV) and disconnecting stage (Stage V).

#### 2.1.1. Stage I

For Stage I, JBS is calculated with Equation (5).

(5) $$\tau \left(x\right)=\frac{F\alpha }{\pi D}\frac{\cosh\left(\alpha x\right)}{\sinh\left(\alpha L\right)}$$

where *F*: loading force of rock bolts; *α*: an elastic coefficient; and *L*: grouting length.

#### 2.1.2. Stage II

For Stage II, JBS is split into two parts: the elastic part and the plastic weakening part. For the elastic part, JBS is calculated with Equation (6):

(6) $$\tau \left(x\right)=\tau_{p}\frac{\cosh\left(\alpha x\right)}{\sinh\left(\alpha \left(L-a_{w}\right)\right)}$$

where *ω*: a plastic coefficient; and *a_w_*: length of the plastic weakening part.

For the plastic weakening part, JBS can be calculated with Equation (7).

(7) $$\begin{array}{l} \tau \left(x\right)=-\tau_{p}\left[\begin{array}{l} \frac{\omega }{\alpha }\tanh\left(\alpha \left(L-a_{w}\right)\right)\cos\left(\omega \left(L-a_{w}\right)\right)-\\ \sin\left(\omega \left(L-a_{w}\right)\right) \end{array}\right]\sin\left(\omega x\right)\\ +\tau_{p}\left[\begin{array}{l} \frac{\omega }{\alpha }\tanh\left(\alpha \left(L-a_{w}\right)\right)\sin\left(\omega \left(L-a_{w}\right)\right)+\\ \cos\left(\omega \left(L-a_{w}\right)\right) \end{array}\right]\cos\left(\omega x\right) \end{array}$$

#### 2.1.3. Stage III

For Stage III, JBS is split into three parts: the elastic part, plastic weakening part and plastic disconnecting part. For the elastic part, JBS is calculated with Equation (8):

(8) $$\tau \left(x\right)=\tau_{p}\frac{\cosh\left(\alpha x\right)}{\cosh\left(\alpha (L-a_{w}-a_{f})\right)}$$

where *a_f_*: length of the plastic disconnecting part.

For the plastic weakening part, JBS is calculated with Equation (9).

(9) $$\begin{array}{l} \tau \left(x\right)=-\tau_{p}\left[\begin{array}{l} \frac{\omega }{\alpha }\tanh(\alpha (L-a_{w}-a_{f}))\cos(\omega (L-a_{w}-a_{f}))-\\ \sin(\omega (L-a_{w}-a_{f})) \end{array}\right]\sin\left(\omega x\right)\\ +\tau_{p}\left[\begin{array}{l} \frac{\omega }{\alpha }\tanh(\alpha (L-a_{w}-a_{f}))\sin(\omega (L-a_{w}-a_{f}))+\\ \cos(\omega (L-a_{w}-a_{f})) \end{array}\right]\cos\left(\omega x\right) \end{array}$$

For the plastic disconnecting part, JBS is calculated with Equation (10).

(10) $$\tau \left(x\right)=\tau_{r}$$

#### 2.1.4. Stage IV

For Stage IV, JBS is split into two parts: the plastic weakening part and plastic disconnecting part. For the plastic weakening part, JBS is calculated with Equation (11).

(11) $$\tau \left(x\right)=\tau_{r}\frac{\cos\left(\omega x\right)}{\cos\left(\omega a_{w}\right)}$$

For the plastic disconnecting part, JBS is calculated with Equation (12).

(12) $$\tau \left(x\right)=\tau_{r}$$

#### 2.1.5. Stage V

For Stage V, JBS is calculated with Equation (13).

(13) $$\tau \left(x\right)=\tau_{r}$$

Table 2 summarises parameters that are used in this modelling process.

### 2.2. Experimental Validation

**Figure 2:**
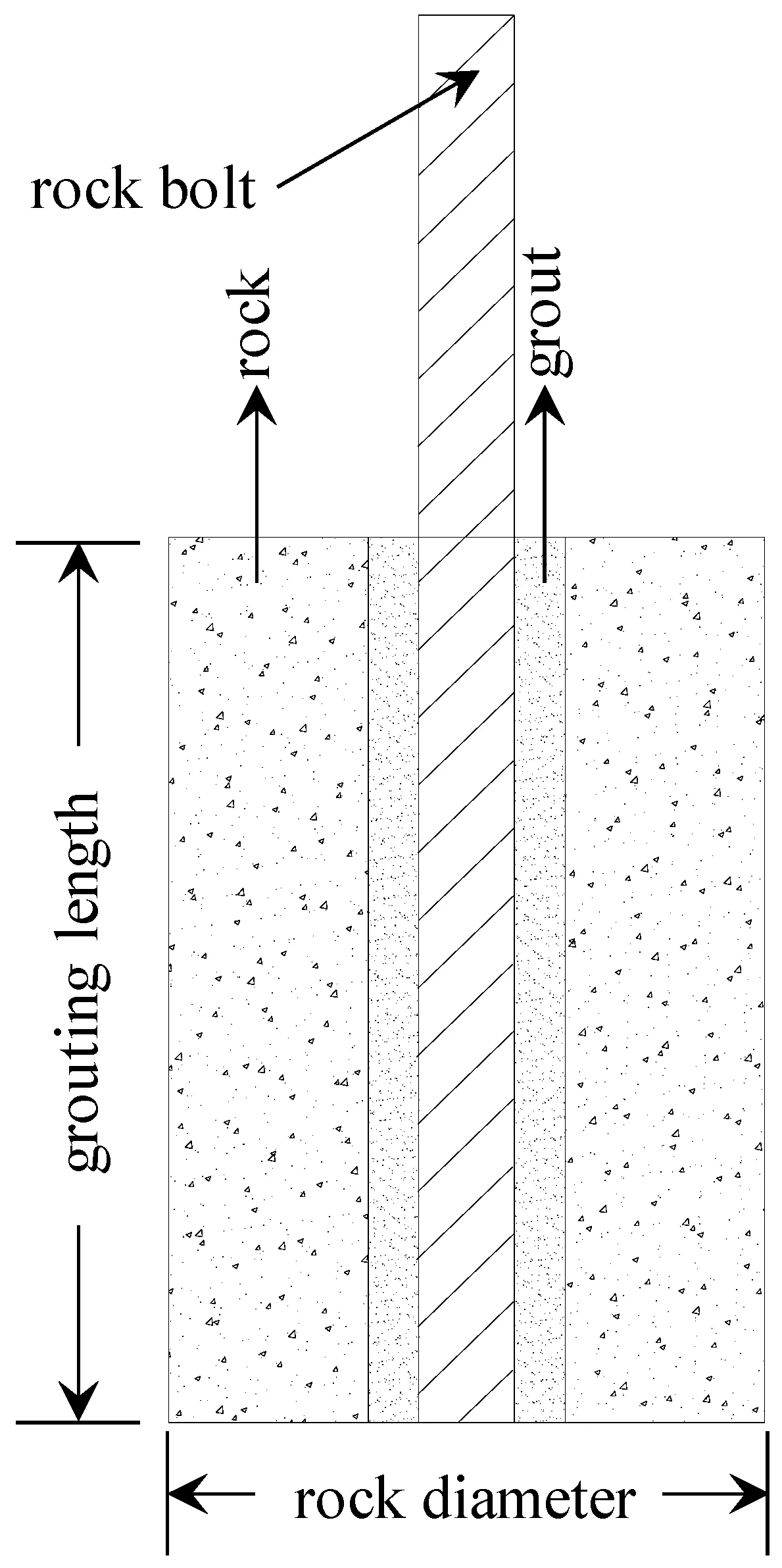
The adopted experimental test method to determine the anchorage force.

To validate the accuracy of the above analytical deducing process, calculated results were compared with experimental results obtained by Rajaie [18]. Specifically, Rajaie [18] conducted multiple anchorage tests on fully grouted bolts. Tested bolts had a diameter of 15.2 mm. A cementitious grout was used to bond bolts with artificial rock mass. The thickness of the grout annulus is 17.9 mm. The thickness of the cylindrical artificial rock blocks is 99.5 mm. Seven different bonded lengths are used, ranging from 150 mm to 700 mm. The experimental test method is shown in Figure 2.

To simulate these anchorage tests, *τ_p_* is set to 5.84 MPa, *τ_r_* is set to 2.81 MPa, *δ_p_* is set to 9.9 mm, and *δ_r_* is set to 19.9 mm. Results show that the analytical results are consistent with the experimental results [16]. This confirmed the accuracy of the above analytical deduction process.

## 3. Parametric Study

To evaluate the influence of grouting conditions on JBS distribution, an analytical anchorage test scenario was created. Physical and mechanical properties of rock bolting systems are listed in Table 3.

As for the joint between rock bolts and grout, its mechanical properties were listed below. *τ_p_* was set to 3 MPa, *τ_r_* was set to 0.5 MPa, *δ_p_* was set to 1 mm, and *δ_r_* was set to 5 mm.

Then, rock bolts were pulled axially from the host rock mass. During the pulling process, JBS was recorded to analyse its distribution under different grouting conditions. To evaluate the influence of parameters on JBS distribution, JBS distributions from Stage I to Stage V were plotted by varying the corresponding parameters. For Stage I and Stage II, the JBS distribution at the end of each stage was plotted. For Stage III, the JBS distribution at the point of the maximum anchorage force was plotted. For Stage IV and Stage V, the JBS distribution at the beginning of each stage was plotted.

### 3.1. The Influence of Joint Strength

**Figure 3:**
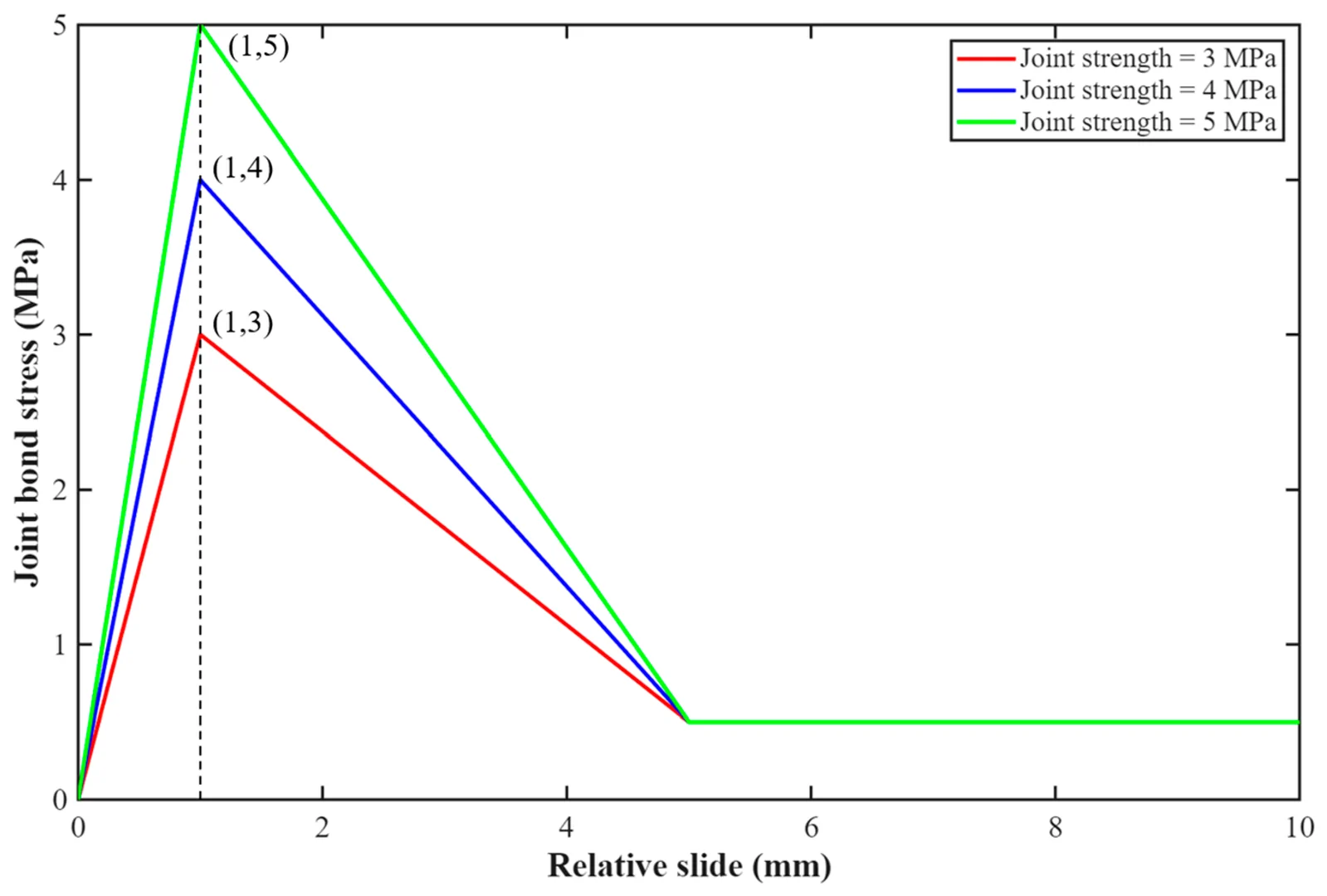
Variation process of joint bond stress when joint strength increased from 3 MPa to 5 MPa.

Joint strength is largely dependent on grouting conditions, surface geometry of rock bolts and surrounding stress. To study the influence of joint strength on JBS distribution, three different values were used for joint strength, ranging from 3 MPa to 5 MPa (Figure 3).

**Figure 4:**
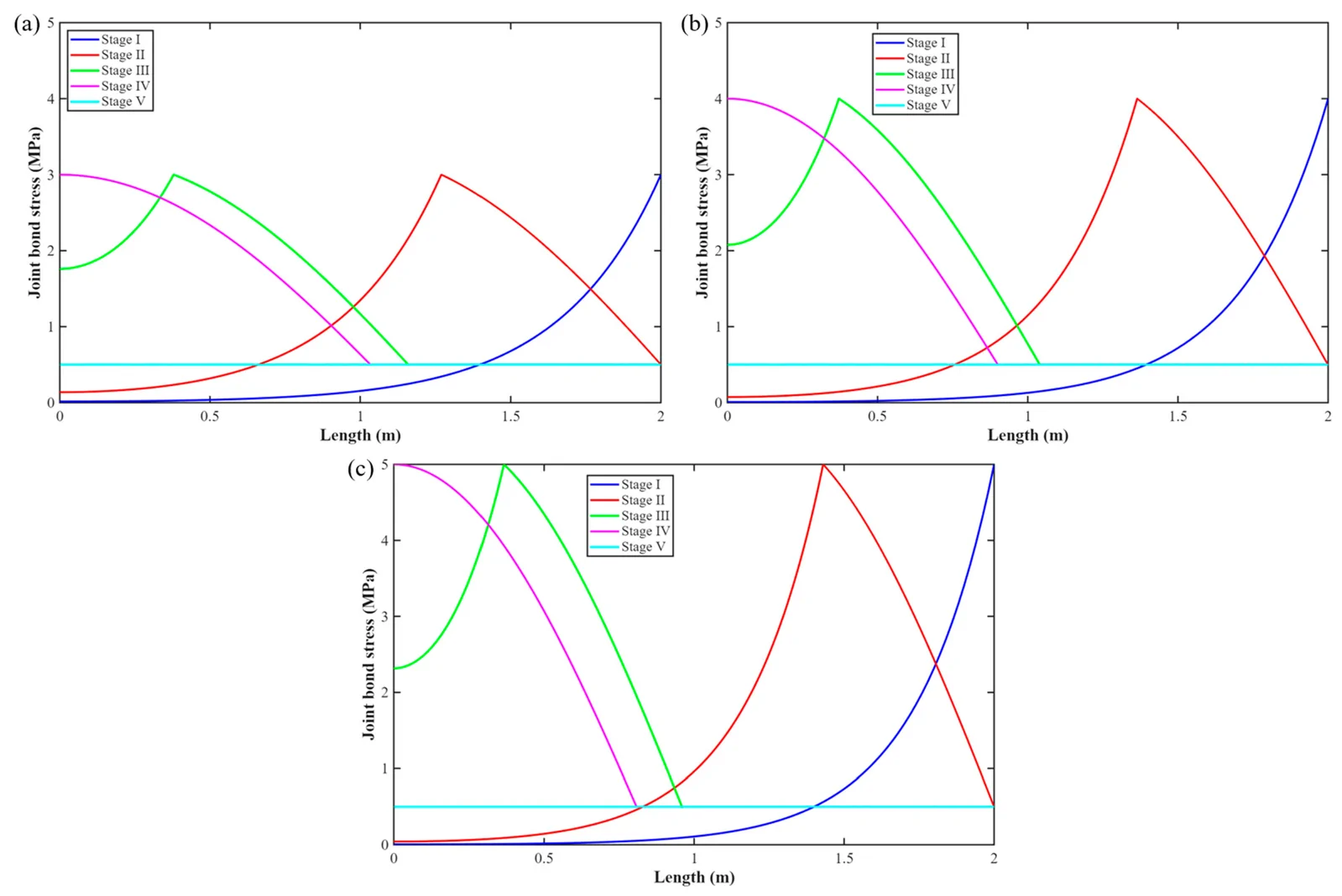
Evolution process of joint bond stress during the loading process of rock bolts: (**a**) joint bond stress distribution when joint bond strength was 3 MPa; (**b**) joint bond stress distribution when joint bond strength was 4 MPa; (**c**) joint bond stress distribution when joint bond strength was 5 MPa.

The JBS distribution was shown in Figure 4. Apparently, joint strength played a crucial role in determining the JBS distribution. With joint strength increasing, the cross-sectional area below JBS distribution lines was apparently larger. Moreover, for Stage I–Stage IV, the maximum JBS increased to 5 MPa. However, joint strength had no influence on Stage V. This shows that in Stage V, JBS distribution was identical when joint strength varied. Moreover, in Stage V, JBS remained at 0.5 MPa along the entire length of the rock bolts. It should be mentioned that although joint strength increased from 3 MPa to 5 MPa, it had no influence on JBS distribution stages. When joint strength varied, the whole joint consistently underwent five different stages.

**Figure 5:**
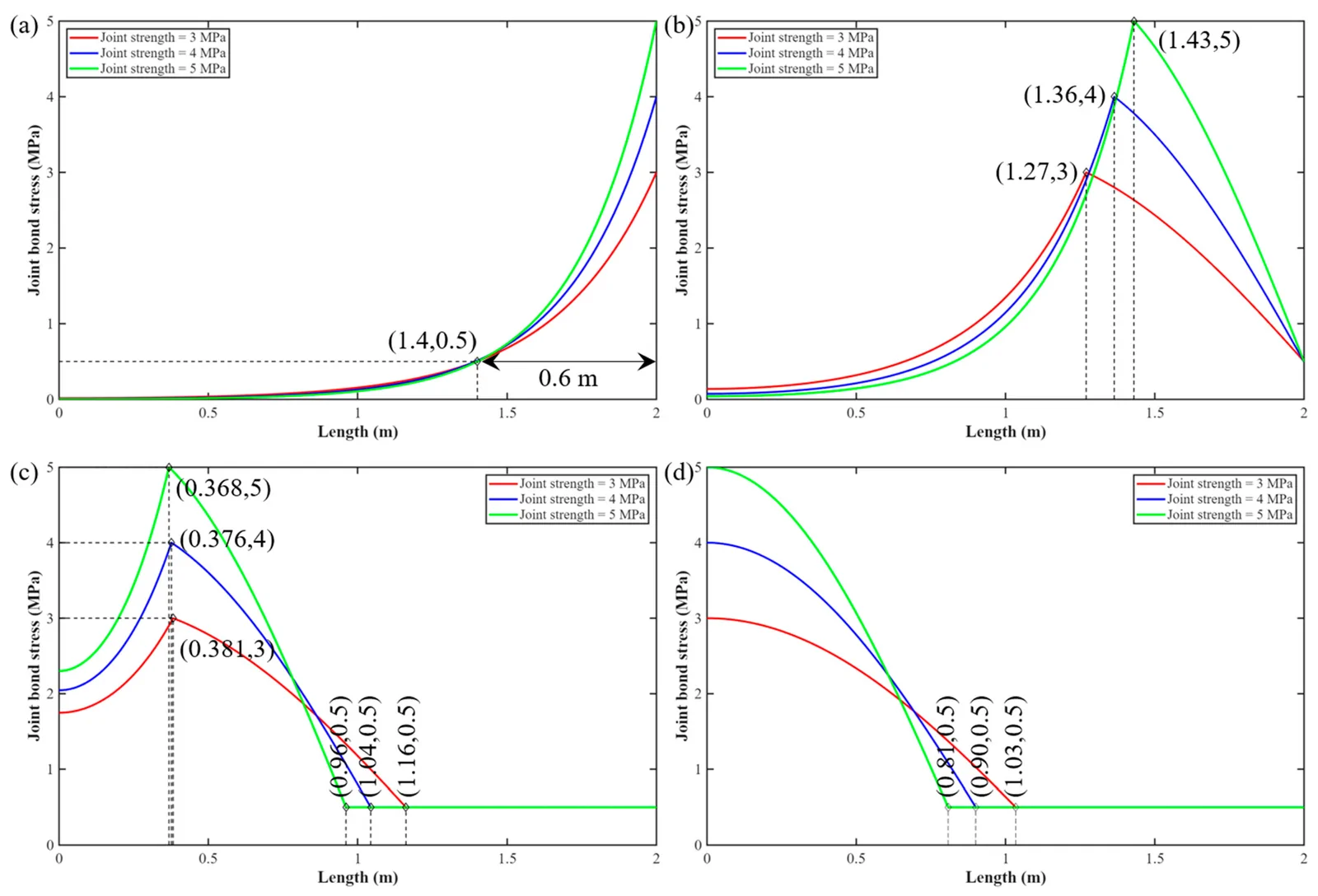
Comparison of joint bond stress distribution when joint strength increased from 3 MPa to 5 MPa: (**a**) Stage I; (**b**) Stage II; (**c**) Stage III; (**d**) Stage IV.

To further quantitatively investigate the influence of joint strength on JBS distribution, the JBS distribution for Stage I–Stage IV was further studied (Figure 5).

Figure 5a shows that in Stage I, JBS decreased exponentially from the external end of rock bolts to the internal end. It also shows that when the buried depth of rock bolts was less than 0.6 m, joint strength exerted a crucial influence on the JBS distribution. With joint strength increasing from 3 MPa to 5 MPa, along the external 0.6 m, JBS was apparently larger. However, once the buried depth was deeper than 0.6 m, JBS was almost identical. This indicated that before Stage I ended, there was an influence range for JBS. When the buried depth of rock bolts was less than the influence range, the joint strength crucially influenced the JBS distribution. However, once the buried depth of rock bolts was larger than this influence range, further improvement in joint strength had a marginal influence on the JBS distribution. For the scenario in Figure 5a, the influence range was 0.6 m.

Figure 5b shows that when Stage II ended, the joint strength exerted a crucial influence on the position where it occurred. In this case, when the joint strength was 3 MPa, the maximum JBS was located 1.27 m away from the internal end of rock bolts. When the joint strength was 4 MPa, the maximum JBS was located 1.36 m away from the internal end of rock bolts. When the joint strength was 5 MPa, the maximum JBS was located 1.43 m away from the internal end of rock bolts. Therefore, results showed that when Stage II ended, if the joint strength was small, it was likely to propagate into a deeper position. By contrast, when Stage II ended, if the joint strength was large, it was likely to propagate within a shallow range.

Figure 5c shows the JBS distribution when the maximum anchorage force occurred in Stage III. It was interesting to see that although the joint strength was different, the position where the maximum JBS occurred was almost consistent. When the joint strength was 3 MPa, the maximum JBS was located 0.381 m away from the internal end. When the joint strength was 4 MPa, the maximum JBS was located 0.376 m away from the internal end. When the joint strength was 5 MPa, the maximum JBS was located 0.368 m away from the internal end. Therefore, although joint strength increased by 66.7%, the difference in the position where the joint strength occurred was just 3.5%. Nevertheless, the joint strength exerted a crucial influence on the disconnected length. In this case, when the joint strength was 3 MPa, the disconnected section ranged from 1.16 m to 2 m. Then, the disconnected length was 0.84 m. When the joint strength was 4 MPa, the disconnected section ranged from 1.04 m to 2 m. Then, the disconnected length was 0.96 m. When the joint strength was 5 MPa, the disconnected section ranged from 0.96 m to 2 m. Then, the disconnected length was 1.04 m. Therefore, with joint strength increasing, the disconnected length also increased when the maximum anchorage force occurred in Stage III.

Figure 5d shows that in Stage IV, the joint strength had a crucial influence on the decreasing trend of JBS in the weakening section. For the weakening section, the larger the joint strength, the deeper the decreasing trend of JBS. Moreover, the joint strength also influenced the disconnected length. When the joint strength was 3 MPa, the disconnected section ranged from 1.03 m to 2 m. Then, the disconnected length was 0.97 m. When the joint strength was 4 MPa, the disconnected section ranged from 0.90 m to 2 m. Then, the disconnected length was 1.10 m. When the joint strength was 5 MPa, the disconnected section ranged from 0.81 m to 2 m. Then, the disconnected length was 1.19 m. It indicated that the larger the joint strength, the longer the disconnected length. This was consistent with analysis results obtained from Figure 5c.

Results showed that joint bond strength had a significant influence on the JBS distribution. As for the reason, it was inferred that after joint strength increased, the grout had more ability to restrain rock bolts. Along the joint between rock bolts and grout, in both the elastic section and the weakening section, the joint should overcome more resistance due to increased joint strength. This led to the apparent redistribution of JBS.

**Figure 6:**
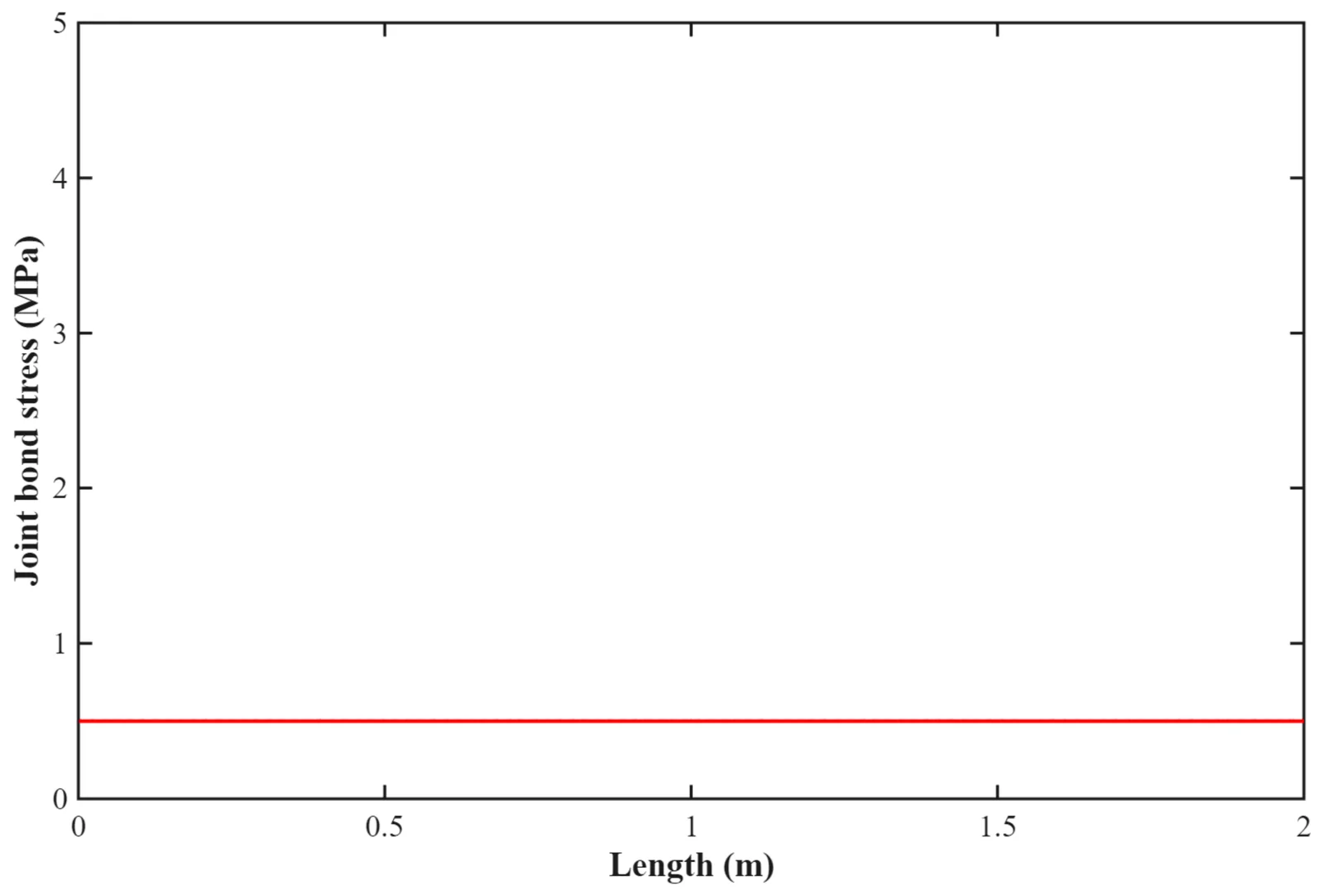
Joint bond stress distribution remained identical when joint strength varied from 3 MPa to 5 MPa.

Nevertheless, the joint strength had no influence on the JBS distribution state for Stage V. Figure 6 shows that although the joint strength increased from 3 MPa to 5 MPa, the JBS distribution state remained identical in Stage V.

### 3.2. The Influence of Joint Remaining Strength

**Figure 7:**
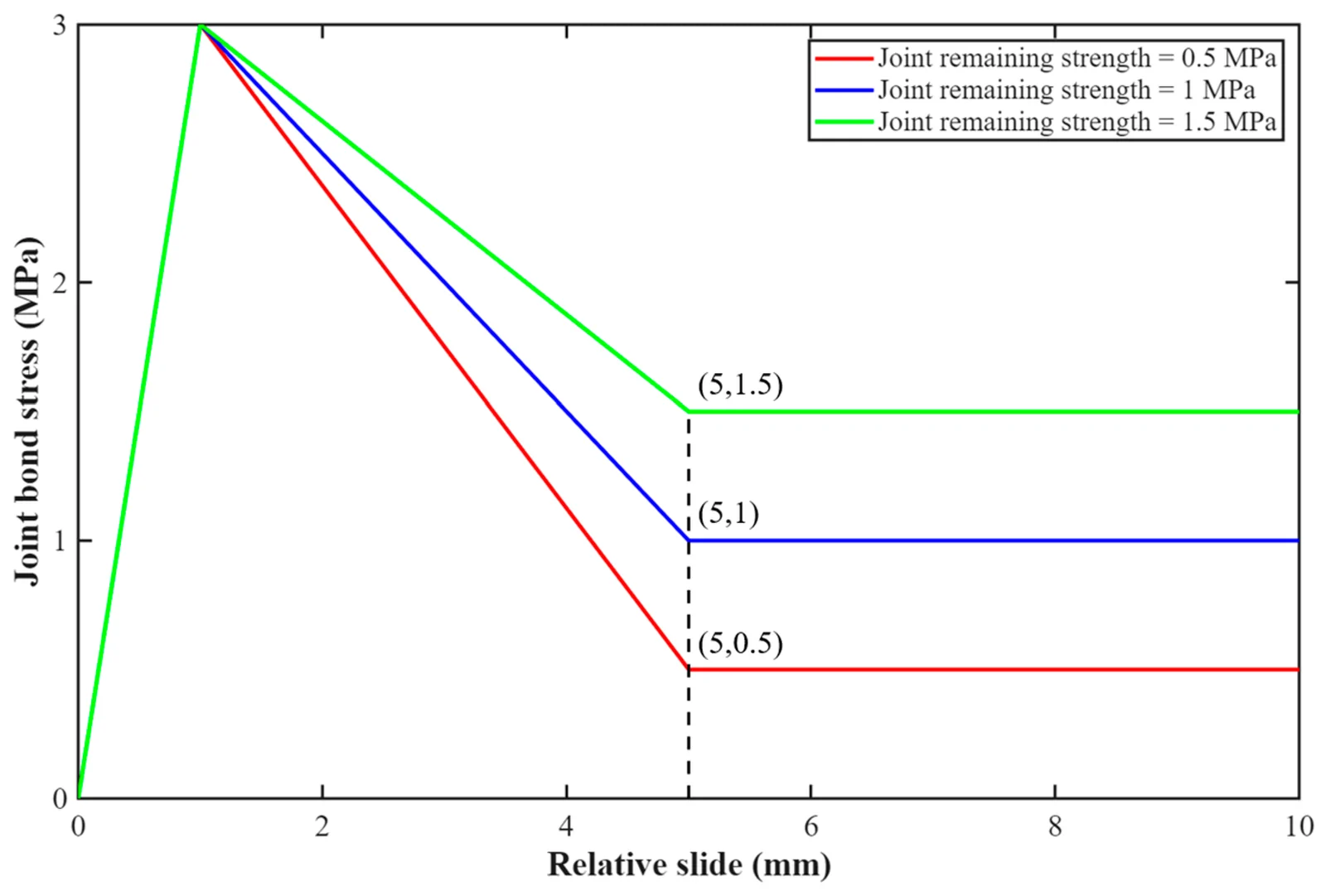
Variation process of joint bond stress when joint remaining strength increased from 0.5 MPa to 1.5 MPa.

To study the influence of joint remaining strength on the JBS distribution, the joint remaining strength ranged from 0.5 MPa to 1.5 MPa (Figure 7). As for other parameters, they were identical to the input values listed in Section 3.

**Figure 8:**
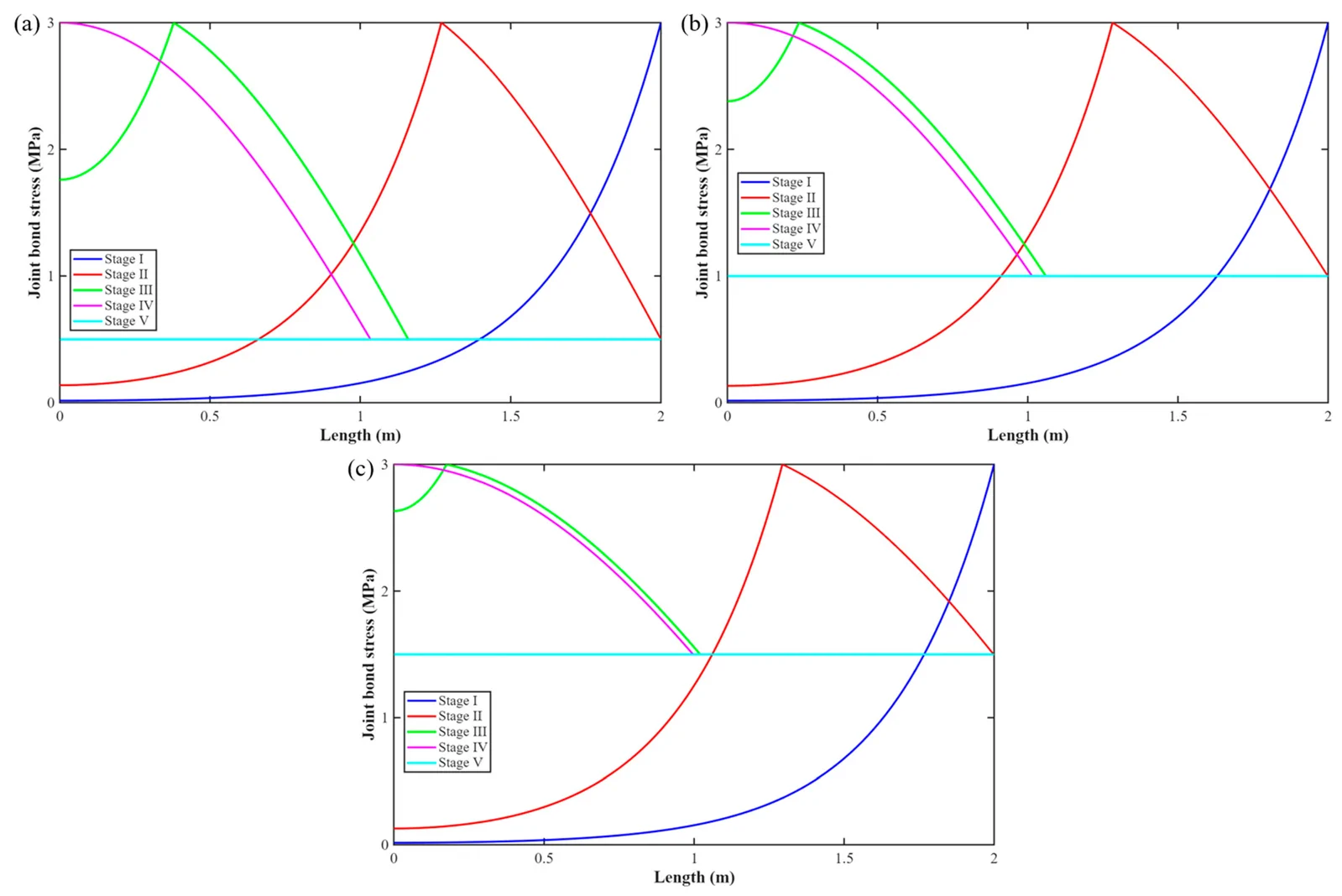
Evolution process of joint bond stress during the loading process of rock bolts: (**a**) joint bond stress distribution when joint remaining strength was 0.5 MPa; (**b**) joint bond stress distribution when joint remaining strength was 1 MPa; (**c**) joint bond stress distribution when joint remaining strength was 1.5 MPa.

Then, rock bolts were loaded, and the JBS distribution was shown in Figure 8. Apparently, the joint remaining strength had no influence on the JBS distribution in Stage I. However, it had an influence on the JBS distribution in Stage II–Stage V.

**Figure 9:**
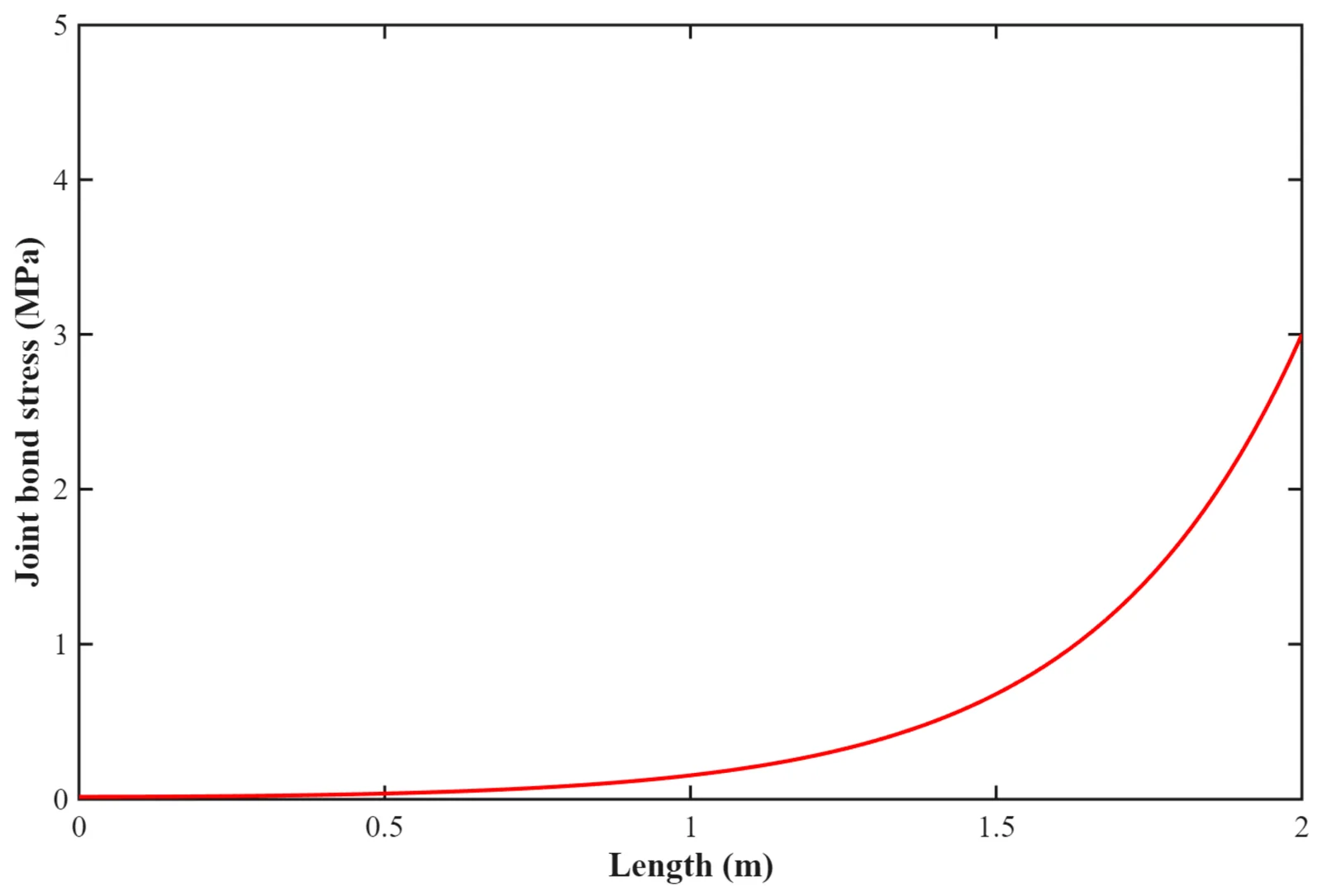
Joint bond stress distribution remained identical when joint remaining strength increased from 0.5 MPa to 1.5 MPa.

Figure 9 shows the JBS distribution in Stage I when the joint remaining strength varied. It shows that when the joint remaining strength increased from 0.5 MPa to 1.5 MPa, the JBS distribution was identical when Stage I ended. Therefore, after rock bolts were loaded, the joint remaining strength had no influence on the JBS distribution when the joint deformed elastically.

**Figure 10:**
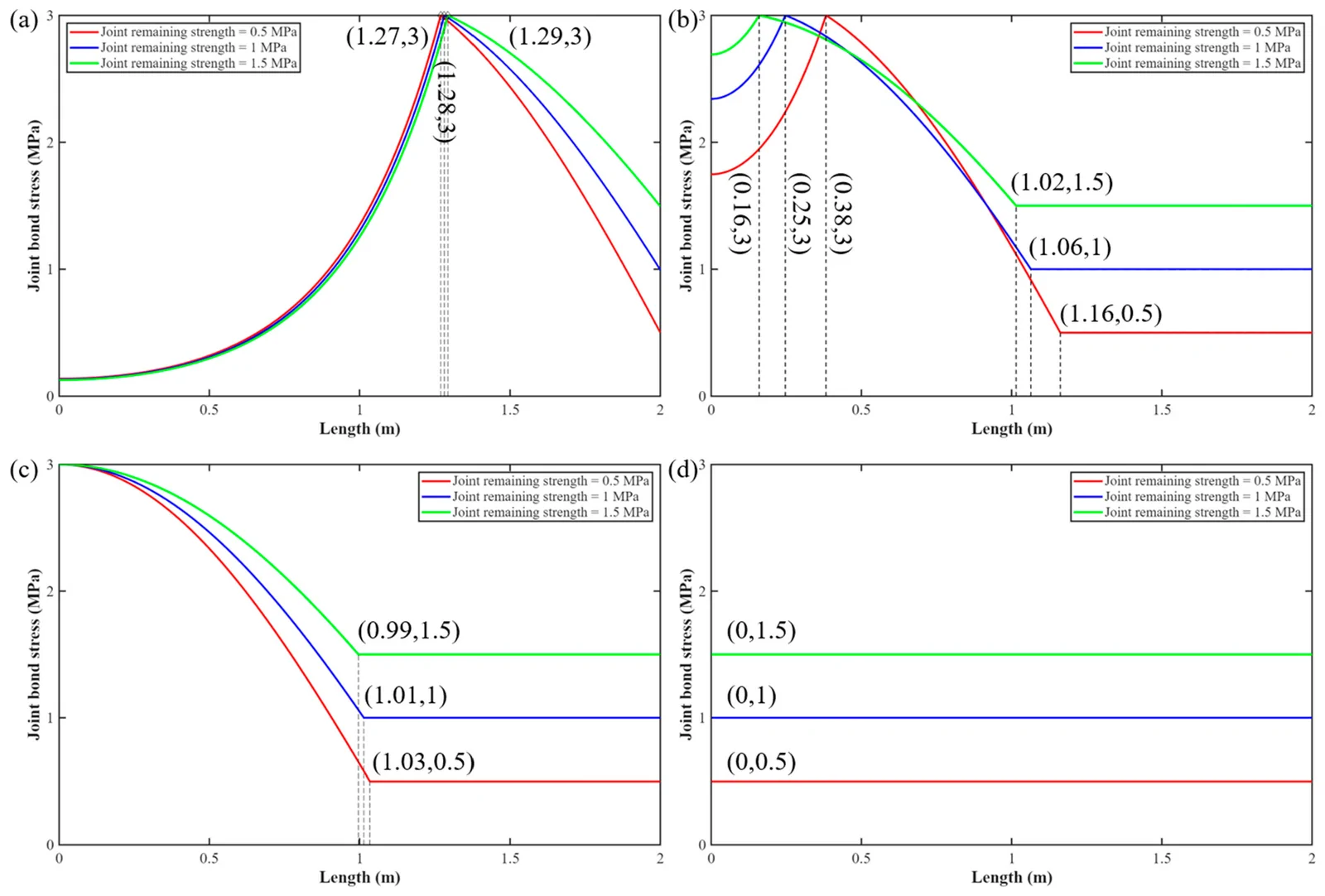
Comparison of joint bond stress distribution when joint remaining strength increased from 0.5 MPa to 1.5 MPa: (**a**) Stage II; (**b**) Stage III; (**c**) Stage IV; (**d**) Stage V.

To further study the influence of joint remaining strength on the JBS distribution, for Stage II–Stage V, the JBS distribution was plotted (Figure 10).

Figure 10a shows that when Stage II ended, the joint remaining strength had almost no influence on the position where the maximum bond stress occurred. When the joint remaining strength was 0.5 MPa, the maximum JBS was located 1.27 m away from the internal end. When the joint remaining strength was 1 MPa, the maximum JBS was located 1.28 m away from the internal end. When the joint remaining strength was 1.5 MPa, the maximum JBS was located 1.29 m away from the internal end. The relative difference in the position where the maximum JBS occurred was just 1.57%. This indicated that the joint remaining strength had a marginal influence on the position where the maximum JBS occurred when Stage II ended.

Figure 10b shows the JBS distribution when the maximum anchorage force occurred. It shows that the joint strength had an apparent influence on the position where the maximum JBS occurred. When the joint remaining strength was 0.5 MPa, the maximum JBS was located 0.38 m away from the internal end. When the joint remaining strength was 1 MPa, the maximum JBS was located 0.25 m away from the internal end. When the joint remaining strength was 1.5 MPa, the maximum JBS was located 0.16 m away from the internal end. Therefore, in Stage III, when the maximum anchorage force occurred, the maximum JBS became closer to the internal end of rock bolts with joint strength increasing. Moreover, in Stage III, with joint strength increasing, the disconnected length also increased. When the joint remaining strength was 0.5 MPa, the disconnected section ranged from 1.16 m to 2 m. Then, the disconnected length was 0.84 m. When the joint remaining strength was 1 MPa, the disconnected section ranged from 1.06 m to 2 m. Then, the disconnected length was 0.94 m. When the joint remaining strength was 1.5 MPa, the disconnected section ranged from 1.02 m to 2 m. Then, the disconnected length was 0.98 m. Therefore, larger joint remaining strength led to larger disconnected length when the maximum anchorage force occurred in Stage III.

Figure 10c shows that when Stage IV started, the joint remaining strength had a marginal influence on the disconnected length. When the joint remaining strength was 0.5 MPa, the disconnected section ranged from 1.03 m to 2 m. Then, the disconnected length was 0.97 m. When the joint remaining strength was 1 MPa, the disconnected section ranged from 1.01 m to 2 m. Then, the disconnected length was 0.99 m. When the joint remaining strength was 1.5 MPa, the disconnected section ranged from 0.99 m to 2 m. Then, the disconnected length was 1.01 m. Results indicated that with the joint remaining strength increasing from 0.5 MPa to 1.5 MPa, there was only a small difference of 4% in the disconnected length when Stage IV started.

Figure 10d shows the JBS distribution in Stage V. Apparently, in Stage V, JBS along the whole joint equalled the joint remaining strength. Moreover, joint remaining strength had no influence on the JBS distribution form along the joint. With joint remaining strength increasing, only the magnitude of JBS increased.

Results showed that compared with Stage I and Stage II, the influence of joint remaining strength on the JBS distribution was more apparent in Stage III, Stage IV and Stage V. The reason for this was inferred to be that when the joint between rock bolts and grout disconnected, the joint’s disconnecting behaviour started making a difference to JBS. After the joint remaining strength increased, the joint had more ability to resist movement of rock bolts. This led to the apparent JBS distribution in Stage III, Stage IV and Stage V.

### 3.3. The Influence of Relative Slide at Joint Remaining Strength

**Figure 11:**
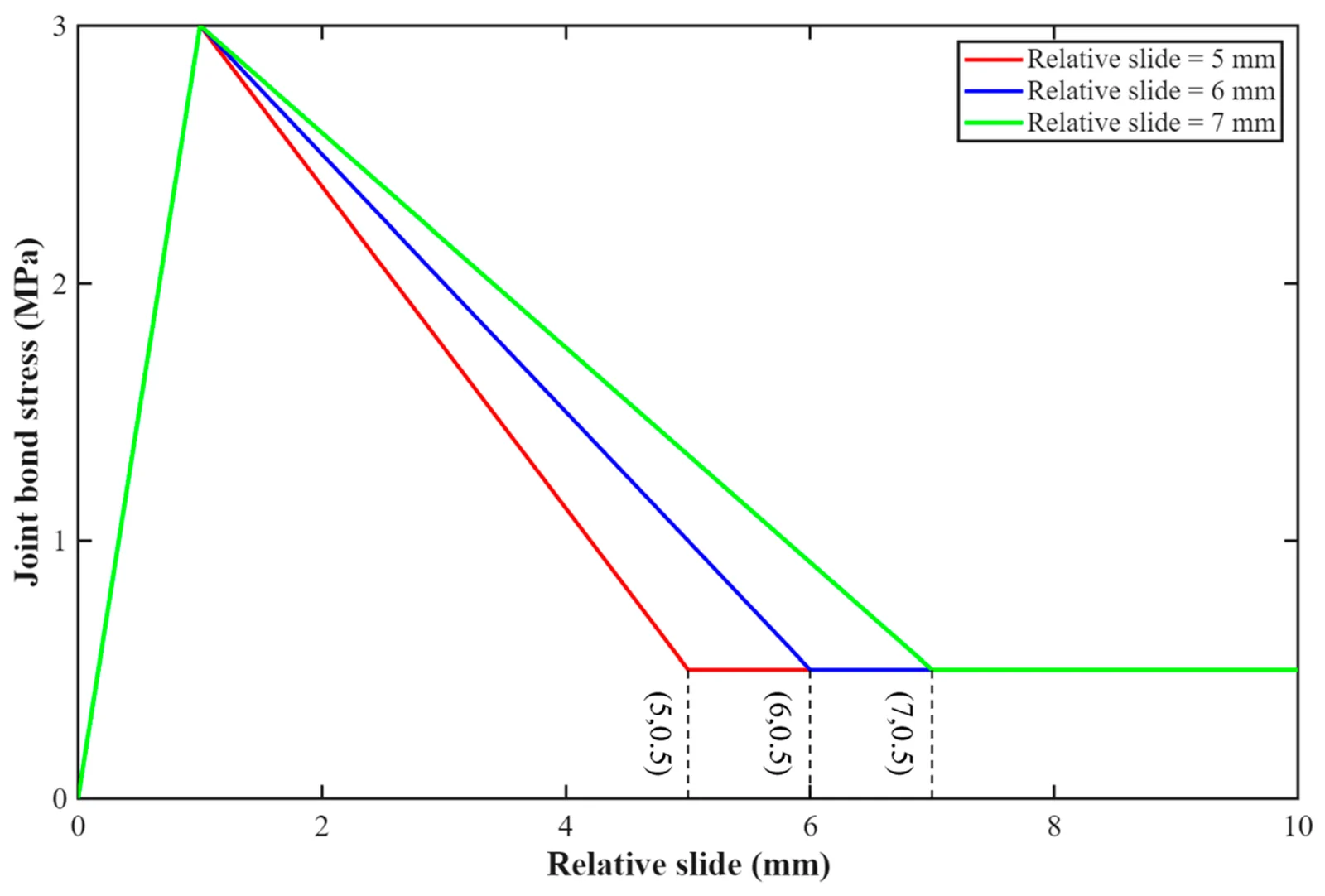
Variation process of joint bond stress when relative slide at joint remaining strength increased from 5 mm to 7 mm.

To study the influence of relative slide at joint remaining strength on the JBS distribution, the relative slide at joint remaining strength was set to three values, ranging from 5 mm to 7 mm (Figure 11).

**Figure 12:**
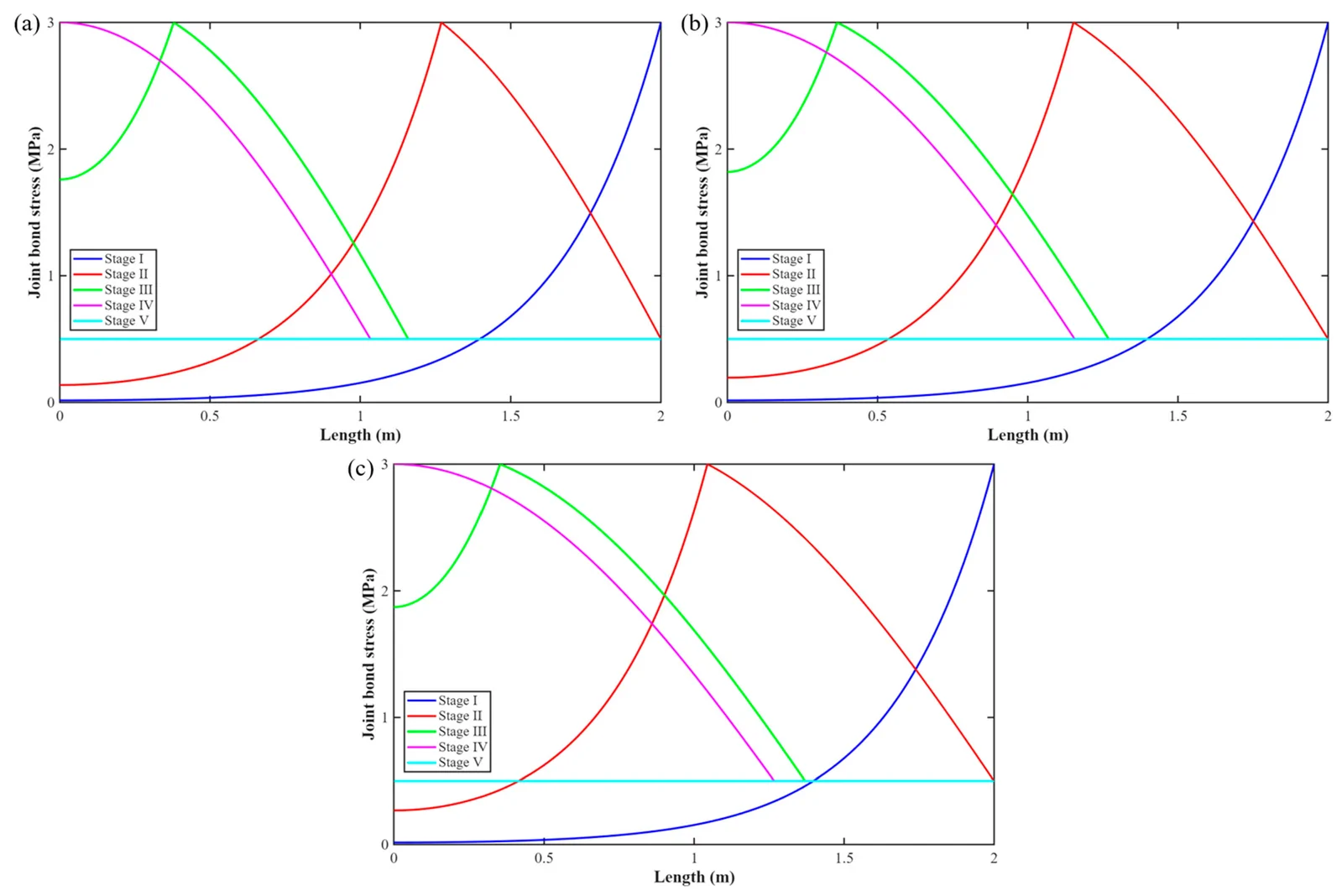
Evolution process of joint bond stress during loading process of rock bolts: (**a**) joint bond stress distribution when relative slide at joint remaining strength was 5 mm; (**b**) joint bond stress distribution when relative slide at joint remaining strength was 6 mm; (**c**) joint bond stress distribution when relative slide at joint remaining strength was 7 mm.

Figure 12 shows the JBS distribution when the relative slide at joint remaining strength varied. It shows that, compared with joint strength and joint remaining strength, relative slide at joint remaining strength had a slightly smaller influence on JBS distribution.

In this case, when Stage I ended, the JBS distribution was identical. Therefore, relative slide at joint remaining strength had no influence on the JBS distribution when Stage I ended.

**Figure 13:**
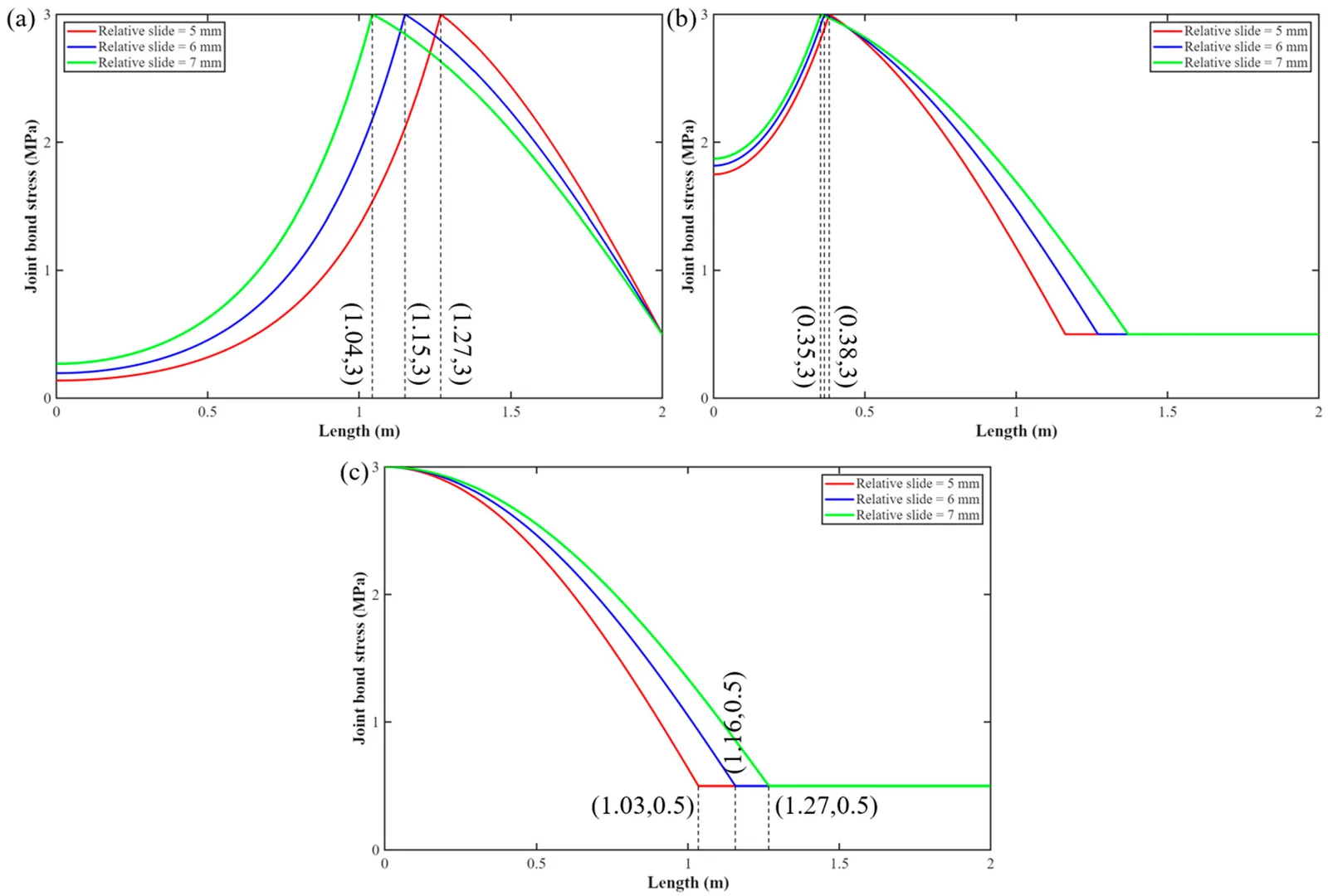
Comparison of joint bond stress distribution when relative slide at joint remaining strength increased from 5 mm to 7 mm: (**a**) Stage II; (**b**) Stage III; (**c**) Stage IV.

Figure 13 shows the influence of the relative slide at joint remaining strength on the JBS distribution in Stage II–Stage IV. It shows that the relative slide at joint remaining strength made a difference in the JBS distribution in these three stages.

Figure 13a shows that the relative slide at joint strength exerted an influence on the JBS distribution state when Stage II ended. With the relative slide at joint remaining strength increasing, the propagation speed of the maximum JBS increased. It means that as the relative slide at the joint remaining strength increased, the distance between the maximum JBS and the internal end decreased. When the relative slide at joint remaining strength was 5 mm, the maximum JBS was located 1.27 m away from the internal end. When the relative slide at joint remaining strength was 6 mm, the maximum JBS was located 1.15 m away from the internal end. When the relative slide at joint remaining strength was 7 mm, the maximum JBS was located 1.04 m away from the internal end. Therefore, larger relative slide led to a smaller distance between the maximum JBS and the internal end of rock bolts.

This phenomenon was also reflected in Stage III. In Stage III, when reaching the maximum anchorage force, the relative slide at joint remaining strength also influenced the position where the maximum JBS occurred, as shown in Figure 13b. Moreover, as the relative slide at joint remaining strength increased, the propagating speed of the maximum JBS increased. When the relative slide at joint remaining strength was 5 mm, the distance between the maximum JBS and the internal end was 0.38 m. When the relative slide at joint remaining strength was 6 mm, the distance between the maximum JBS and the internal end was 0.37 m. When the relative slide at joint remaining strength was 7 mm, the distance between the maximum JBS and the internal end was 0.35 m. Apparently, larger relative slide at joint remaining strength led to a smaller distance between the maximum JBS and the internal end. However, it should be noted that in Stage III, this influence was quite small.

Figure 13c shows the influence of relative slide at joint remaining strength on the JBS distribution in Stage IV. Apparently, the relative slide at joint remaining strength exerted a crucial influence on disconnected length. With increasing relative slide at joint remaining strength, the disconnected length decreased. When the relative slide at joint remaining strength was 5 mm, the disconnected section ranged from 1.03 m to 2 m. Then, the disconnected length was 0.97 m. When the relative slide at joint remaining strength was 6 mm, the disconnected section ranged from 1.16 m to 2 m. Then, the disconnected length was 0.84 m. When the relative slide at joint remaining strength was 7 mm, the disconnected section ranged from 1.27 m to 2 m. Then, the disconnected length was 0.73 m. Therefore, a larger relative slide at joint remaining strength led to a shorter disconnected length.

For Stage V, the JBS distribution was identical. Therefore, the relative slide at joint remaining strength had no influence on the JBS distribution in this stage.

Compared with other parameters, the relative slide at joint remaining strength had less influence on the JBS distribution. As for the reason, it was inferred that when reaching the relative slide at joint remaining strength, the geometric mismatch between rock bolts and grout was damaged. Then, the joint became less sensitive to the relative slide. Consequently, the relative slide at joint remaining strength had a marginal influence on JBS distribution.

For the above three parameters studied in this section, the joint strength was the primary influencing parameter that determined the JBS distribution. Compared with the joint remaining strength, it had more influence on the JBS distribution form, which finally determined the maximum anchorage force of rock bolts. When the joint strength increased from 3 MPa to 4 MPa, increasing by 33%, the maximum anchorage force increased by 14%. By contrast, when the joint remaining strength increased from 0.5 MPa to 1 MPa, increasing by 100%, the maximum anchorage force only increased by 22%. Moreover, compared with the relative slide at joint remaining strength, the joint strength can influence the JBS distribution in four stages. By contrast, the relative slide at joint remaining strength can only influence the JBS distribution in three stages.

The joint remaining strength was the secondary influencing parameter, and the relative slide at joint remaining strength was the least significant influence parameter. This was because, when compared with the relative slide at joint remaining strength, the joint remaining strength can influence more stages of JBS distribution during the loading process of rock bolts. Moreover, the influence of the relative slide at joint remaining strength on the JBS distribution was quite slight.

## 4. Discussion

The JBS distribution is largely determined by the mechanical properties of rock bolts, grout and host rock mass. Moreover, a proper JBS distribution is beneficial for improving the bonding capacity of rock bolts. However, in previous research, more attention was paid to the bonding capacity of rock bolts [19,20,21]. By contrast, much less work has been conducted to study the JBS distribution in rock reinforcement systems. This is probably because of the following reasons. First, it is much easier to evaluate the bonding capacity of rock bolts in experimental tests and analytical studies. To realise this, researchers just need load cells and displacement transducers to record the force and displacement of rock bolts [22]. In an analytical study, researchers just need to deduce equations to calculate the force and displacement of rock bolts [23]. By contrast, if researchers need to evaluate the JBS distribution in rock reinforcement systems, strain gauges should be attached to the joint between rock bolts and grout [24]. This makes the experimental test process quite complicated. In an analytical study, to evaluate the JBS distribution in rock reinforcement systems, researchers should analyse the interaction between rock bolts and grout. Moreover, JBS should be analytically deduced along the rock bolt length [15]. This also makes the deduction process quite complicated.

Analytical results showed that there was an apparent JBS propagation phenomenon. Specifically, at the joint between rock bolts and grout, during the loading process of rock bolts, JBS propagated from the external end of rock bolts to the internal end. This phenomenon was previously revealed by experimental test results. Specifically, Rong et al. [25] conducted anchorage tests on rock bolts. During testing, strain gauges were attached to the rock bolt surface to record tensile stress distribution in rock bolts. Based on the obtained tensile stress distribution, the bond stress can be analysed. Results showed that after rock bolts were loaded, the maximum bond stress generally propagated from the external end of rock bolts to the internal end. Therefore, the analytically calculated results generally agreed with the experimental test results obtained by Rong et al. [25]. This further confirmed the accuracy of the analytical modelling results.

As an improvement based on previous research, this study considered the sliding process of the joint between rock bolts and grout. Then, the JBS distribution can be studied. Based on this concept, the influence of different parameters on the JBS distribution was studied, including joint strength, joint remaining strength and relative slide at joint remaining strength. Results showed that the mechanical properties of the joint apparently affected the JBS distribution. Moreover, the mechanical properties of the joint influenced the maximum JBS propagation process and propagation speed in rock reinforcement systems. This indicates that, based on this study, researchers can adopt it to calculate the JBS distribution. Then, based on the calculated results, researchers can optimise the mechanical properties of the joint to further improve the bonding capacity of rock bolts.

Previous research indicated that by improving joint strength, the bonding capacity and the maximum anchorage force of rock bolts can be improved [26]. However, the exact reason was not explained in detail. In this study, when joint strength increased, the JBS distribution state was calculated during the loading process of rock bolts. Results showed that after joint strength increased, the following consequences were obtained. First, when rock reinforcement systems deformed elastically, JBS around the external end of rock bolts increased significantly. This led to the result that around the external end of rock bolts, rock bolts had increased ability to resist deformation of rock mass. Secondly, in Stage II, the joint underwent elastic behaviour and weakening behaviour simultaneously. In Stage II, after joint strength increased, the maximum JBS propagated more slowly towards the internal end of rock bolts. Consequently, around the internal end of rock bolts, further elastic deformation can be provided by the joint with longer length. When the maximum anchorage force was obtained, with joint strength varying, the position where the maximum JBS occurred was almost identical. This indicates that in this case, along the joint, the elastic length was almost equal. However, in the elastic section, larger joint strength led to JBS increasing to a high limit. Moreover, because of larger joint strength, JBS increased throughout the whole elastic section. Consequently, this led to higher anchorage force of rock bolts.

The above analysis was also applicable to the parameter of joint remaining strength. After the joint remaining strength increased, the maximum anchorage force of rock bolts could also be improved. The reason for this is explained below. After a disconnection occurred along the joint, increased joint remaining strength led to a larger frictional force between rock bolts and grout in the disconnecting section. This contributed to an increase in the maximum anchorage force of rock bolts.

This study is beneficial for a better understanding of the anchorage mechanism of rock bolts. Based on this analytical model, users can analyse the JBS distribution when reaching the maximum anchorage force. Then, along the joint between rock bolts and grout, the disconnecting position and disconnecting length can be determined. Based on calculated results, users can optimise grout properties to prevent disconnecting failure. Then, this is beneficial for preventing failure of rock bolting systems for in situ cases. Therefore, this study has apparent practical applications. For example, this study revealed that when joint strength increased, the joint had greater resistance to overcome in the elastic section and elastic-weakening section. This led to an increase in the anchorage force of rock bolts. Therefore, for in situ applications, users can improve grout strength to optimise joint strength. Then, the overall anchorage performance of rock bolting systems can be enhanced.

However, this current study still has certain shortcomings. In this study, a three-equation model was used to calculate JBS variation. This three-equation model was a simplified method. Experimental tests on short-encapsulated rock bolts showed that JBS might not have a linear relationship with relative slide [4]. In fact, after rock bolts were loaded, at the initial loading period, JBS increased linearly with relative slide. However, once the joint around the external end yielded, the increasing rate of JBS started dropping. Then, a non-linear relationship existed between JBS and relative slide. After JBS reached a peak, there was an apparent non-linear relationship between JBS and relative slide in the post-peak period. It is admitted that once JBS dropped to a certain value, JBS was likely to become stable. However, this stable JBS cannot be sustained all the time. This is because, with further slide between rock bolts and grout, the joint was likely to become smoother. Because of this, once relative slide increased to a critical limit, the joint resistance may even be removed. Based on the above analysis, the three-equation model may not exactly reflect the bonding behaviour of the joint. In this study, the authors adopted the three-equation model because it was relatively easier to incorporate the three-equation model into rock reinforcement systems and calculate the corresponding JBS distribution state in rock reinforcement systems. Nevertheless, it is necessary to propose new bond-slip models to exactly capture the non-linear mechanical behaviour of joints.

Second, the analytical model was validated with limited experimental test results. Based on this shortcoming, more anchorage tests should be conducted to further validate the accuracy of this analytical model.

Third, this study assumes that the grout material followed relatively simpler mechanical behaviour. However, for in situ cases, the mechanical behaviour of grout materials may be quite complicated. Therefore, this study can be further extended to consider complicated behaviour of the grout material.

## 5. Conclusions

In this study, a three-equation model was used to depict the mechanical behaviour of the joint between rock bolts and grout. This three-equation model was incorporated into rock reinforcement systems. Then, the interaction between rock bolts, grout and rock mass was studied. After that, the JBS distribution was analysed. The following conclusions were obtained.

(1) For rock reinforcement systems, after rock bolts were loaded, the JBS propagation process can be basically divided into five different stages, including Stage I–Stage V. From Stage I to Stage V, the maximum JBS propagated from the external end of rock bolts to the internal end.
(2) Joint strength crucially affected the JBS distribution in rock reinforcement systems. When joint strength increased, JBS apparently increased around the external end of rock bolts when Stage I ended. However, joint strength had no apparent influence on the JBS distribution around the internal end of rock bolts. When reaching the maximum anchorage force, JBS in the elastic section around the internal end can be crucially improved with joint strength increasing. This benefited the improvement in bonding capacity of rock bolts.
(3) Joint remaining strength had no influence on the JBS distribution in Stage I. For Stage II, it only had a slight influence on the JBS distribution. After joint remaining strength increased, when reaching the maximum anchorage force, disconnected length and corresponding frictional resistance increased. This benefited improving the bonding capacity of rock bolts.
(4) Compared with joint strength and joint remaining strength, relative slide at joint remaining strength had a relatively slight influence on JBS distribution. When relative slide at joint remaining strength varied, there was no apparent difference in the JBS distribution in Stage I and Stage V. In Stage II, with the relative slide at joint remaining strength increasing, the maximum JBS propagated faster from the external end of rock bolts to the internal end. This influence was also reflected in Stage III. However, in Stage III, this influence became much gentler.
(5) This study adopts an analytical method to evaluate the JBS distribution in rock bolting systems when grout properties change. Therefore, the contribution of this study is that, based on this analytical method, users can better understand the influence of grout properties on the JBS distribution. For practical applications, this study provides scientific guidance for engineers to vary grout properties to prevent failure of rock bolting systems.

## Figures and Tables

**Table 1:** Comparison between different bond-slip models.

Bond-Slip Models	Differences	Assumptions	Applicability
Two-equation model	This model is only composed of two parts, including the linear elastic part and the linear weakening part.	With the relative slide increasing to a limit, joint bond stress (JBS) can be assumed to disappear.	This model is applicable for the case when the joint between rock bolts and grout fails; the frictional resistance at the joint can be neglected.
Three-equation model	This model is composed of three parts, including the linear elastic part, linear weakening part and horizontal disconnecting part.	When there is relative slide between rock bolts and grout, the joint between rock bolts and grout undergoes elastic, weakening and disconnecting behaviour.	This model considers elastic, weakening and disconnecting behaviour of the joint between rock bolts and grout. Therefore, this model is applicable for most rock bolting scenarios.
Non-linear model	This model can be simulated with a single equation. From the start to the end, it is fully non-linear.	It is assumed that there is full non-linear behaviour of the joint between rock bolts and grout after rock bolting systems are loaded.	This model is applicable for the case where the joint between rock bolts and grout deforms fully non-linearly.

**Table 2:** Summary of parameters used in this modelling process.

Parameters	Physical Meaning	Units	Data Source
*D*	diameter of rock bolts	m	measured based on the test scenario
*τ_p_*	joint strength	Pa	measured based on the test scenario
*δ_p_*	relative slide at joint strength	m	measured based on the test scenario
*τ_r_*	joint remaining strength	Pa	measured based on the test scenario
*α*	an elastic coefficient	m^−1^	obtained from the intermediate calculation process
*ω*	a plastic coefficient	m^−1^	obtained from the intermediate calculation process
*L*	grouting length	m	measured based on the test scenario
*E_b_*	Young’s modulus of rock bolts	Pa	measured based on the test scenario
*E_g_*	Young’s modulus of grout	Pa	measured based on the test scenario
*E_m_*	Young’s modulus of rock mass	Pa	measured based on the test scenario
*v_g_*	Poisson’s ratio of grout	1	measured based on the test scenario
*t*	grout thickness	m	measured based on the test scenario
*b*	rock mass thickness	m	measured based on the test scenario

**Table 3:** Physical and mechanical properties of rock bolting systems.

*D* (mm)	*E_b_* (GPa)	*L* (m)	*E_g_* (GPa)	*v_g_*	*t* (mm)	*E_m_* (GPa)	*b* (mm)
22	200	2	20	0.1	2	30	10

## Data Availability

The original contributions presented in this study are included in the article. Further inquiries can be directed to the corresponding author.
